# Gender impacts the post-exercise substrate and endocrine response in trained runners

**DOI:** 10.1186/1550-2783-5-7

**Published:** 2008-02-26

**Authors:** Lisa M Vislocky, P Courtney Gaine, Matthew A Pikosky, William F Martin, Nancy R Rodriguez

**Affiliations:** 1 Department of Nutritional Sciences, 3624 Horsebarn Hill Road Ext. Unit 4017, University of Connecticut, Storrs, Connecticut, USA

## Abstract

**Background:**

Although several studies have investigated gender differences in the substrate and endocrine responses during and following endurance exercise, few have studied sex differences during a more prolonged recovery period post endurance exercise. The purpose of this study was to compare and characterize the endocrine and substrate profiles of trained male and female adult runners during the three-and-a-half hour recovery period from an endurance run.

**Methods:**

After consuming a euenergetic diet (1.8 g·kg^-1^·d^-1 ^protein, 26% fat, 58% carbohydrates, 42.8 ± 1.2 kcal/kg body weight) for 8 days, blood was collected from trained male (n = 6, 21 yrs, 70 kg, 180 cm, 9% body fat, VO_2peak _78.0 ± 3.4 mL·kg FFM^-1^·min^-1^) and female (n = 6, 23 y, 66 kg, 170 cm, 29% body fat, VO_2peak _71.6 ± 4.5 mL·kg FFM^-1^·min^-1^) endurance runners at rest and during recovery from a 75 min run at 70% VO_2peak_. Circulating levels of glucose, lactate, free fatty acids (FFAs), insulin, cortisol, growth hormone (GH), and free insulin-like growth factor I (IGF-I) were measured.

**Results:**

During the recovery period, females experienced increases in glucose, lactate and insulin while no changes were noted in men (*P *< 0.05). Males experienced increases in GH and decreases in IGF-I levels respectively (*P *< 0.05) while no changes were observed in females. FFA levels increased during recovery from endurance exercise, but changes were not different between genders.

**Conclusion:**

These data further document gender differences in substrate and endocrine changes during a prolonged recovery period following endurance exercise. Future studies are needed to evaluate the effect of differing diets and nutritional supplements on these gender-specific post-exercise substrate and endocrine differences.

## Background

Fat and carbohydrates are the primary fuel sources used by muscle during aerobic exercise [1]. Intramyocellular triglycerides (IMTGs) and plasma free fatty acids (FFAs) are the primary sources of fat while muscle glycogen and blood glucose are the major sources of carbohydrates provided to working muscle. Endogenous protein is not a major fuel source during exercise [2]. While protein oxidation will increase as exercise intensity increases [3] and/or individuals become glycogen depleted [4], its contribution to total energy production remains minimal. In general, the relative contribution of each substrate to energy production is complicated by many factors such as exercise intensity, duration, subject training status, diet, and gender.

Despite 25 years of research and over 20 studies conducted, the existence of gender differences in macronutrient metabolism during endurance exercise continues to be debated. Discrepancies in the research to date are likely the result of various methodological variables such as 1) lack of adequate control measurements to determine a true effect of exercise [5], 2) failure to control for hormonal changes (i.e. estrogen, progesterone) related to the menstrual cycle phase [6,7], 3) failure to adequately match for training status between genders [8], 4) failure to control diet prior to measurements [9,10], and 5) differences in methodologies employed to determine differences [5]. Studies controlling for most, if not all, of these variables collectively suggest that gender differences exist even when females are in the follicular phase (d 1 – 11) of the menstrual cycle [11-13]. During the early segment of this phase, estrogen and progesterone concentrations are lowest providing an opportunity for comparisons to be made with males [14].

Previous studies have identified gender differences in the substrate, hormone and catecholamine response to exercise. Specifically, during mild-to-moderate intensity endurance exercise lasting up to two hours, females appear to oxidize proportionately more fat and males more carbohydrate and protein [5,15,16]. These differences are not routinely observed at higher exercise intensities [17,18] and are evident regardless of training status [5,19]. Additionally, there are a few published studies reporting that during submaximal endurance exercise males experience greater increases in catecholamine and growth hormone (GH) concentrations [5], while females exhibit greater increases in glucose and insulin [15]. However, it is difficult to make firm conclusions regarding gender specific responses to exercise because data are conflicting and few studies have employed similar designs.

Not only are the gender-specific metabolic outcomes not clear, but the potential mechanisms responsible for such gender-specific responses during aerobic exercise are unknown. Researchers have attributed observed differences to a combination of the complex hormonal response to exercise stress compounded by the cyclic changes in circulating estrogen and progesterone concentrations females experience throughout the menstrual cycle. In addition, gender differences in the autonomic nervous system (ANS) response to endurance exercise have been identified recently and appear to be a major physiological difference driving these disparities [20].

The aim of the present study was to identify gender differences in the substrate and hormone changes occurring during a three-and-a-half-hour recovery period following an endurance exercise bout of moderate intensity in trained runners. While few studies have reported on the prolonged endocrine and substrate responses following exercise, even fewer have reported on these following a moderate intensity run in trained males and females. In the current study, we attempted to control for potential confounding factors known to impact exercise metabolism by 1) matching subjects based on training volume (running ≥ 56 km·wk^-1^) and fitness level (VO_2peak _values), 2) controlling for menstrual cycle phase (follicular) when studying female subjects, and 3) feeding subjects a controlled euenergetic diet (1.8 g·kg^-1^·d^-1 ^protein, 30% fat, 26% fat) 8 days prior to the exercise intervention and substrate and endocrine assessments. We hypothesized that gender disparities would exist in post-exercise endocrine and substrate profiles during three-and-a-half hours of recovery from a prolonged run of moderate intensity.

## Methods

### Experimental design

This investigation consisted of two independent studies in men and women for which all experimental conditions were identical. In both studies, subjects consumed a euenergetic diet providing 1.8 g·kg^-1^·day^-1 ^protein (PRO), 30% of total calories as fat, and 58% carbohydrate (CHO) for 8 days prior to the assessment of hormone and substrate changes during recovery from a 75 min run at 70% VO_2peak_.

### Subjects

Following project approval by Institutional Review Board at the University of Connecticut, male (n = 6) and female (n = 6) athletes (aged 18–30 y; running ≥ 56 km·wk^-1^, VO_2peak _> 45 mL·kg^-1^·min^-1 ^and > 50 mL·kg^-1^·min^-1^, for females and males, respectively) were recruited to participate in each study. Participants provided a complete medical history, training log, and a 3 day diet record. Women were excluded if they were pregnant, taking oral contraceptives, or reporting irregular menses (cycles < 28 or > 35 d). Men and women who reported metabolic or cardiovascular abnormalities, gastrointestinal disorders, vegetarian dietary practices, and the use of nutritional/sports supplements or anabolic steroids were ineligible to participate. Subjects did not resistance train during or for one month prior to participating the study. Informed, written consent was obtained from all participants. Females were eumenorrheic and studied during the early follicular phase (days 1–7) of their menstrual cycles. Previous menstrual history was used to estimate desired study day. Menstrual cycle phase was confirmed by self-report of most recent menses and determination of plasma estradiol concentrations on the study day.

### Preliminary assessments

Baseline data collection included an assessment of aerobic capacity (VO_2peak_), anthropometry (height and weight), body composition, resting energy expenditure (REE-indirect calorimetry), three day diet records, and training logs.

### Peak oxygen uptake

Peak oxygen uptake (VO_2peak_) testing was determined via breath by breath analysis of expired gases during testing using an open circuit respiratory apparatus (MedGraphics CPX/D, Medical Graphics Corporation, St. Paul, MN) on a treadmill (Quinton MedTrack ST55, Bothell, W.A.) [21]. During the VO_2peak _test, the participant ran while the treadmill grade was increased by 2% every two minutes until volitional exhaustion. Test end points included: an overall rating of perceived exertion ≥ 18; a respiratory exchange ratio > 1.1; achievement of age predicted maximal heart rate; and/or inability to maintain pace. Not all volunteers achieved a true VO_2max _as defined by a plateau in oxygen consumption despite increasing workloads. Therefore, VO_2peak _is used to represent their maximal effort as defined by the highest value recorded (mL·kg BW^-1^·min^-1^) during the test.

### Anthropometric measures

Height and weight were measured on a balance beam scale (Health-o-meter Inc., Bridgeview, IL) to the nearest 0.5 kg and 0.5 cm, respectively.

### Body composition determination

For male subjects, percent body fat was estimated using hydrostatic weighing. Body composition was calculated from body density according to equations by Brozek et al [22]. Body composition was determined in women with a whole-body dual-energy X-ray absorptiometry (DXA) scan performed with a bone densitometer, DPX-MD (LUNAR Corp. Madison, WI). Although assessments were made with different methodologies, Hicks et al has reported no significant difference in body fat values obtained using hydrodensitometry versus DXA [23].

### Resting energy expenditure

REE was measured using open-circuit indirect calorimetry (MedGraphics CPX/D, Medical Graphics Corporation, St. Paul, MN) for determination of REE for calorie needs before study participants began the diet intervention. Subjects drove or were driven to the metabolic laboratory immediately after waking, were fasted overnight (10 h) and rested quietly 15–20 min before REE was determined over a 20 min period with the subject lying in a quiet, temperature-regulated room.

### Diet records

Three day diet records were collected from all study participants to assess their baseline nutrient intake for energy, carbohydrates, protein, and fat. All dietary records were analyzed using Nutritionist Pro Software™ (First Data Bank, Inc., Version 1.1).

### Training logs

One week training logs were kept by study participants at baseline to determine exercise-related energy needs for meal planning using the American College of Sports Medicine's Compendium of Physical Activities, 2000 [24]. Study volunteers were also asked to keep training logs detailing their daily and weekly running mileage during the study.

### Prestudy diet intervention

Individual energy requirements were determined based on REE measurements, estimates of energy intake from nutrient analysis of three day diet records, and estimates of energy expenditure based on data obtained from training logs. Energy intakes were prescribed to maintain weight throughout each study and were adjusted accordingly if gains or losses of greater than 1% of body wt occurred. Protein intake was set at 1.8 g·kg^-1^·d^-1 ^body wt and fat at 30% of total energy intake, with carbohydrate intake providing the remainder of the calories. These levels of protein, fat, and carbohydrate intake were selected based on recommendations for endurance athletes [25]. All meals and snacks were provided to study participants for 8 days prior to the substrate and endocrine assessment to ensure dietary adaptation had occurred [26]. To document weight maintenance throughout the diet intervention, body wt for all subjects was obtained biweekly, on the same days, and at the same time following an overnight fast and early morning void. Use of alcohol and caffeine was not permitted throughout the study.

Participants ate in a designated dining hall at the University of Connecticut where research assistants were present to weigh and serve the prescribed foods. All food consumed was recorded for later nutrient analyses. All daily menus and baseline three day diet records were analyzed for energy and macronutrient composition using Nutritionist Pro Software™ (First Data Bank, Inc., Version 1.1).

### Exercise study protocol

Subjects trained and competed throughout the study but refrained from exercise the day before the exercise protocol (~36 h). On the study day, volunteers drove or were driven to the Metabolic Assessment Lab at the Department of Nutritional Sciences at ~0600 h, following a 10–12 h overnight fast. A 20-gauge Teflon catheter (7.78 cm, Jelco, Critikon, Tampa, FL) was inserted into an antecubital vein for baseline blood sample collection (REST). Following baseline blood sample collection, participants were moved to the treadmill (Quinton MedTrack ST55, Bothell, WA) for the 75 min run at 70% VO_2peak _(mL·kg BW^-1^·min^-1^). After subjects completed the run, a catheter (3.97 cm) was placed in the contralateral hand which was heated (to 65°C) with a heating pad for the sampling of arterialized blood [27] 30 min post-exercise (POST + 30), 45 min post-exercise (POST + 45), and 210 minutes (3 1/2 h) post-exercise (POST + 210). The indwelling catheter was kept patent with a slow, isotonic saline drip. Runners drank water *ad libitum *during the entire protocol.

### Substrate and hormone analysis

Samples were collected in lithium-heparin and EDTA monovettes (Sarstedt, Newton, NC. Lithium-Heparin: 01.1604.100; EDTA: 01.1605.100), centrifuged (5,000 rpm) for 10 min, and the supernatant frozen at -80°C for later analyses. All blood samples were processed and assayed in duplicate for substrates and hormones as described below.

Glucose and lactate concentrations were determined simultaneously using an automated glucose oxidase method (YSI Model 2300, Yellow Springs, OH). Plasma samples for non-esterified free fatty acid (FFA) determination were assayed using an enzymatic, colorimetric assay (Wako Chemicals, Richmond, VA). Plasma estradiol concentrations were determined in females only using solid phase, double antibody RIA kits (DSL 4400, Diagnostic Systems Laboratories, Webster, TX). Insulin concentrations were determined using a solid-phase, double antibody radioimmunoassay (RIA) (DSL-1600, Diagnostic Systems Laboratories, Webster, TX). Cortisol concentrations were determined using an enzyme immunoassay (EIA) (DSL-10-2000, Diagnostic Systems Laboratories, Webster, TX). Growth hormone (GH) and free insulin-like growth factor I (IGF-I) levels were determined using immunoradiometric (IRMA) assays (Diagnostic System Laboratories, Webster, TX (Growth hormone, DSL-1900; Free-IGF, DSL-9400)).

### Statistical analysis

As noted earlier in the methods, these data were from two independent studies for which the same controls and protocols were used [28,29] which prevented direct comparisons from being made between genders at each time point. However, the overall difference in gender responses was reported. In addition, an analysis of variance (ANOVA) was conducted to evaluate the effect of time post-exercise (REST, POST + 30, POST + 45, POST + 210) on substrate and hormone responses. When statistical significance was observed, a Tukey's *post hoc *analysis was used to identify pairwise differences. The alpha level was set at 0.05. All data were analyzed using SPSS^® ^11.0 Statistical Software (SPSS Inc., Chicago, IL). All data are presented as mean ± SEM.

## Results

### Subject characteristics

Participant characteristics are presented in Table 1. Participants studied were a mix of collegiate runners and serious recreational athletes. Males were taller, weighed more, had a lower percent body fat and body mass index (BMI; kg/m^2^), and ran approximately 14 more miles per week when compared to the females (*P *< 0.05). VO_2peak _values (mL·kg BW^-1^·min^-1^) differed between groups, but when adjusted for FFM (because of differences in body fat between groups), VO_2peak _values were similar between males and females. Subjects maintained baseline weights throughout their respective dietary intervention.

**Table 1:** Baseline subject characteristics for male and female runners.

	**Females**	**Males**
Age (yr)	22.7 ± 0.7	20.6 ± 0.6
Height (cm)	166.9 ± 2.6	180.0 ± 5.0
Height (kg)	66.2 ± 4.7	70 ± 1.2
Body Fat (%)	28.8 ± 1.8	9.0 ± 0.5 #
Body Mass Index (BMI; kg*m^2^)	22.9 ± 1.6	21.6 ± 0.4
VO_2 _(mL*kg FFM^1^*min^1^)	71.6 ± 4.5	78.0 ± 3.4

Values expressed as mean ± SEM. # Methods for determination of % body fat differed for males and females (i.e. hydrodensitometry vs DXA, dual-energy X-ray absorptiometry), respectively. VO_2peak_, peak oxygen consumption; FFM, fat free mass.

### Dietary intervention

Energy intake and macronutrient content remained constant for the duration of the study. Energy intake for females was 2,384 ± 78.6 kcal (36.0 ± 1.2 kcal/kg body weight), 56% carbohydrate (5.1 g·kg^-1^), 19% protein (1.8 g·kg^-1^), and 26% fat, while men consumed 3,463 ± 87 kcal (49.5 ± 1.2 kcal/kg body weight), 60% carbohydrate (7.4 g·kg^-1^), 14% protein (1.78 g·kg^-1^), and 26% fat.

### Substrates

Gender differences existed in the plasma glucose and lactate response to endurance exercise.

### Glucose

**Figure 1:**
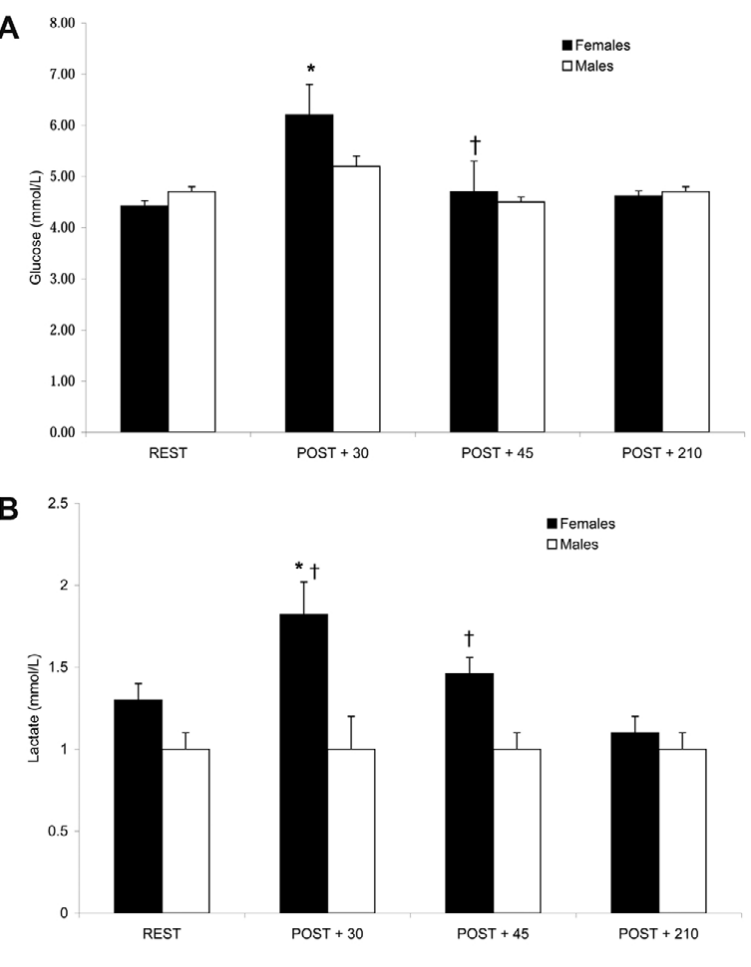
**Plasma glucose (A) and lactate (B) concentrations prior to and during recovery from endurance exercise**. Gender differences existed in glucose and lactate responses post-exercise. Values expressed as mean ± SEM. REST, prior to exercise; POST+30, 30 min post-exercise; POST + 45, 45 min post-exercise; POST + 210, 210 min post-exercise. * Different from REST in females, † Different from POST + 30 in females, # Different from 210 min POST in females, *P *< 0.05.

In females, glucose concentrations increased POST + 30 (*P *< 0.05), but returned to almost resting levels by POST + 45 (*P *< 0.05) while no changes in plasma glucose were noted in males in response to exercise (Fig 1A). (REST, 4.4 ± 0.1 and 4.7 ± 0.1; POST + 30, 6.2 ± 0.6 and 5.2 ± 0.2; POST + 45, 4.7 ± 0.6 and 4.5 ± 0.1; POST + 210, 4.6 ± 0.1 and 4.7 ± 0.1 mmol/L for females and males, respectively).

### Lactate

Similarly, in females, lactate concentrations increased POST + 30 (*P *< 0.05), remained elevated vs POST + 210 at POST + 45, and returned to resting values by POST + 210. (REST, 1.3 ± 0.1 and 1.0 ± 0.1; POST + 30, 1.8 ± 0.1 and 1.0 ± 0.1; POST + 45, 1.5 ± 0.1 and 1.0 ± 0.1; POST + 210, 1.0 ± 0.1 and 1.0 ± 0.1 mmol/L for females and males, respectively). However, plasma lactate did not change in men in response to exercise (Fig 1B).

### FFAs

No gender differences were observed in circulating levels of FFAs. In both sexes, circulating FFA concentrations increased POST + 30 and decreased by POST + 210. (REST, 0.54 ± 0.15 and 0.64 ± 0.06; 30 min POST, 1.45 ± 0.25 and 1.36 ± 0.19; 210 min POST, 0.98 ± 0.10 and 0.95 ± 0.07 mEq/L for females and males, respectively.)

### Hormones

Post-exercise differences in insulin, cortisol, GH and free IGF-I were observed between males and females.

### Estradiol

Plasma estradiol concentrations at rest were 39.2 ± 3.6 pg/mL, confirming that each female participant was studied during the early follicular phase of the menstrual cycle [14].

### Insulin

**Figure 2:**
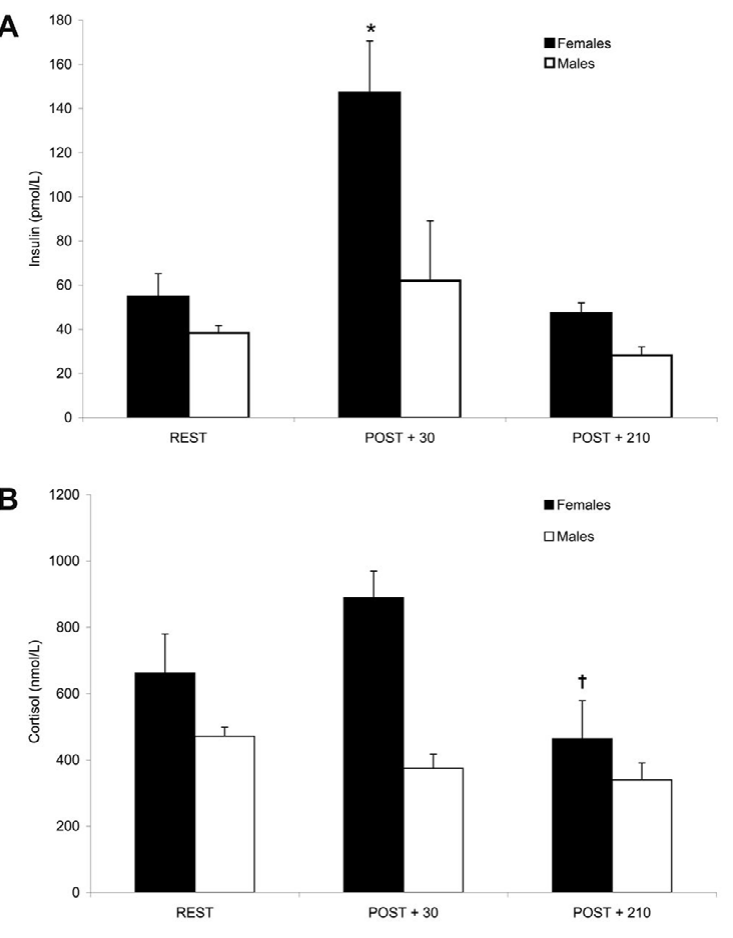
**Plasma insulin (A) and cortisol (B) concentrations prior to and during recovery from endurance exercise**. Gender differences existed in the insulin and cortisol responses post-exercise. Values expressed as mean ± SEM. REST, prior to exercise; POST + 30, 30 min post-exercise; POST + 210, 210 min post exercise). * Different from REST and POST + 210 in females, *P *< 0.01; † Different from POST + 30 in females, *P *< 0.05.

An almost three-fold increase in insulin concentrations was noted in females at POST + 30 (*P *< 0.01) which returned to resting values by POST + 210 (Fig 2A). Insulin levels in males did not change post-exercise. (REST, 54.9 ± 10.3 and 38.2 ± 3.4; POST + 30, 147.1 ± 23.5 and 62.0 ± 27.14; POST + 210 min, 47.4 ± 4.7 and 28.3 ± 3.8 pmol/L for females and males, respectively.)

### Cortisol

There were no differences in cortisol concentrations in males POST + 30. Cortisol concentrations were increased at POST + 30 in all but one female participant, however this trend was not significant (*P *= 0.07). At POST + 210, cortisol concentrations had decreased in females from POST + 30 to REST (*P *< 0.05). There was also a difference in the overall change (magnitude and direction) of the cortisol response at POST + 30 between males and females (*P *< 0.05), i.e. cortisol concentrations increased in females (+ 210.3 ± 118.3 ng/mL) and decreased in males (-95.7 ± 48.1 ng/mL) (Fig 2B). (REST, 661.6 ± 118.2 and 471.0 ± 27.6; POST + 30, 889.7 ± 79.9 and 375.3 ± 42.1; POST + 210, 464.5 ± 113.9 and 339.8 ± 51.2 nmol/L for females and males, respectively.)

### GH

**Figure 3:**
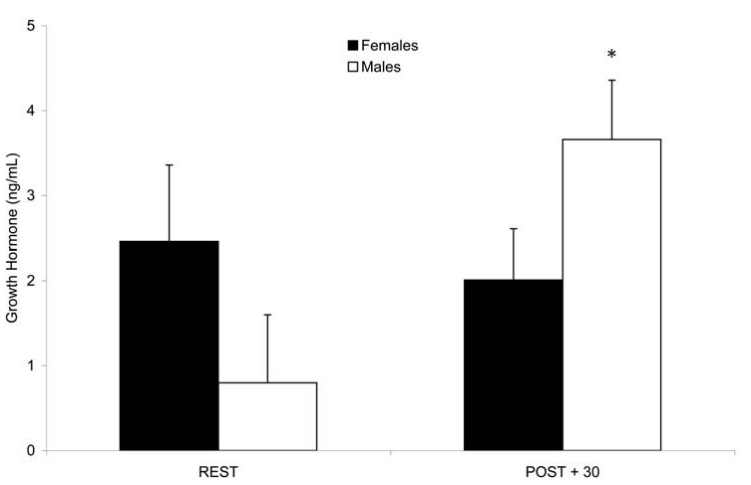
**Plasma growth hormone (GH) concentrations in trained adults prior to and following endurance exercise**. Gender differences existed in the post-exercise GH response. Values expressed as mean ± SEM. REST, prior to exercise; POST + 30, 30 min post-exercise. * Different from REST in males, *P *< 0.05.

A greater than four-fold increase (*P *< 0.05) in GH concentrations was noted in males at POST + 30, while no change was observed in females. There was also a difference in the overall change (magnitude and direction) from REST to POST + 30 in GH concentrations between males and females (*P *< 0.05), i.e. males' levels increased (+ 2.9 ng/mL) while females' values decreased (-0.5 ng/mL) (Fig 3). (REST, 2.5 ± 0.9 and 0.8 ± 0.8; POST + 30, 2.0 ± 0.6 and 3.7 ± 0.7 ng/mL for females and males, respectively.)

### IGF-I

**Figure 4:**
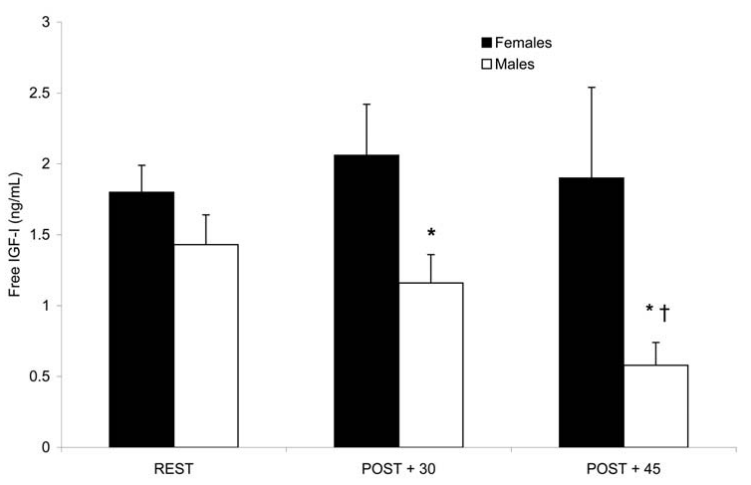
**Plasma free insulin-like growth factor-I (IGF-I) concentrations prior to and following endurance exercise**. Gender differences were observed in the IGF-I response post-exercise. Values expressed as mean ± SEM. REST, prior to exercise; POST + 30, 30 min post-exercise; POST + 45, 45 min post-exercise. * Different from REST in males, *P *< 0.05, † Different from POST + 30 in males, *P *< 0.001.

Free IGF-I concentrations did not change in females in response to exercise. However, IGF-I concentrations decreased in males from REST to POST + 30 (*P *< 0.05) to POST + 45 (*P *< 0.001). IGF-I concentrations in males at POST + 45 were also lower compared to REST (*P *< 0.05) (Fig 4). (REST, 1.8 ± 0.2 and 1.4 ± 0.2; POST + 30, 2.1 ± 0.4 and 1.2 ± 0.2; POST + 45, 1.9 ± 0.6 and 0.6 ± 0.2 ng/mL for females and males, respectively.)

## Discussion

The aim of the present study was to characterize gender differences in the endocrine and substrate responses during a three-and-a-half hour recovery period from an identical bout of endurance exercise in comparably trained male and female runners consuming a diet providing adequate energy and a similar macronutrient composition. The major findings were that substrate and endocrine changes during recovery from a bout of endurance exercise differed between males and females. In particular, we observed a greater absolute increase in glucose, lactate, insulin, and a trend of increased cortisol concentrations post-exercise in females, but little to no change in these parameters for males. Post-exercise, GH concentrations increased and free IGF-I levels decreased in males, while no changes in these hormones were noted in females. No gender differences were observed in circulating FFAs post-exercise. Both males and females experienced significant increases in FFAs following exercise with levels decreasing but still remaining above resting values three-and-a-half hours into recovery. The present study is unique given its focus on identifying gender differences in the metabolic and endocrine profiles during a three-and-a-half hour recovery period following a prolonged run of moderate intensity in endurance trained men and women for whom habitual energy and macronutrient intakes were similar and well-controlled.

In this study, females were studied during the early follicular phase of the menstrual cycle. During this time, estrogen and progesterone levels are lowest and therefore most similar to males [14] and least likely to impact fuel use and metabolic responses following endurance exercise. In humans, (i.e. males and amenorrheic females), only pharmacological doses of E_2, _the most potent and abundant estrogen in the body, have affected substrate utilization (i.e. decreased carbohydrate and amino acid oxidation and increased fat utilization) and/or plasma endocrine and substrate profiles during submaximal aerobic exercise [7,30,31]. Our study design minimized any potential effect of estrogen on the outcome measures by studying females during the follicular phase of the menstrual cycle.

While many studies have investigated gender differences in macronutrient metabolism during submaximal aerobic exercise and reported the simultaneous metabolic and endocrine responses, very few investigations have characterized the substrate and hormone responses during prolonged recovery from endurance exercise. Furthermore, the literature contains only a small number of studies that have investigated whether gender differences exist during the prolonged (i.e. > 90 min) post-exercise response while controlling for potential confounding variables (i.e. menstrual cycle phase, training volume/status, and energy and macronutrient intake prior to measurements). As a result, findings from the present study are discussed in the context of a limited number of investigations [5,15,32].

An earlier investigation from our laboratory [32] outlined the plasma substrate and endocrine profiles of trained male runners during recovery from a two hour run at 65% VO_2max _following an overnight fast. When compared to resting levels, post-exercise (i.e. 20 min following exercise) concentrations of lactate, glucose, FFAs, and GH increased, while those of insulin and cortisol decreased in the earlier study. Similar to these findings, plasma FFAs and GH levels increased during the post-exercise period in the present study. Although little is known about the prolonged post-exercise FFA and GH responses, similarly designed studies have documented increased FFA and GH concentrations in males following endurance exercise [15,33].

In contrast to the findings of our previous work, the present investigation observed no changes POST + 30 compared to REST in males with regards to circulating levels of glucose, insulin, or lactate. These discrepancies might be attributed to the differences in exercise duration (i.e. 120 vs 75 min). Research reporting increased circulating glucose and insulin levels immediately post-exercise have involved aerobic exercise lasting two hours [5]. The post-exercise increases in glucose in the previous study might have been a reflection of a greater reliance on blood glucose versus muscle glycogen as exercise duration increased [5,34] whereas the concurrent increases in insulin likely occurred to offset the increased values. While we observed increases in lactate levels POST + 30 in women, the magnitude of the differences were small and we cannot verify whether these concentrations influenced any of the other parameters measured.

Horton et al [5] conducted one of the few studies reporting hormone and substrate responses during two hours of recovery from a prolonged exercise bout (cycling, 2 h, 40% VO_2max_) in comparably trained males and females with similar fitness capacities (64.4 and 55.5 mL·kg^-1^·min^-1^, respectively). This study was similar to the present investigation in that subjects were matched based on training volume and intensity, fed controlled, euenergetic, diets of similar macronutrient composition for three days prior to measurements, and females were studied during the follicular phase of the menstrual cycle. Post-exercise, these authors observed no gender differences in the substrate responses (i.e. FFAs, lactate), but reported greater glucose concentrations at all time points in males. Gender differences were noted for post-exercise insulin levels which increased in males (presumably to offset simultaneous increases in plasma glucose), but decreased in females. No differences were observed in the post-exercise cortisol responses for males and females. GH and IGF-I were not measured.

Our findings were similar to those of Horton and colleagues in that during recovery from endurance exercise we observed no gender differences in the plasma FFA changes, but identified disparities in the gender response with respect to glucose and insulin. As expected, FFA concentrations increased from REST to POST + 30 in both genders, an observation supported by others [33]. However, we reported increases in glucose and insulin concentrations POST + 30 in only females while Horton et al reported similar changes exclusively in males. Although not statistically significant, the tendency for the concentrations of cortisol, a potent gluconeogenic hormone, to be greater at POST + 30 in women might be of physiological importance and have contributed to the differences observed. We presume insulin levels increased to counteract elevated plasma glucose levels, but it is unclear from our data which occurred first. The differences may ultimately be the result of the differences in the exercise intensity of the cycling bout (2 h, 40% VO_2max_) used by Horton et al compared to the 75 min run at 70% intensity in the present study.

The majority of our findings are also consistent with those reported by Tarnopolsky et al [15] who identified gender differences in the prolonged post-exercise response for plasma glucose, FFAs, insulin, and GH in trained male and female runners completing a 15.5 km run at 65% VO_2max_. The experimental design was similar to the present study in that subjects were fed a controlled diet prior to measurements (3 d), males and females were matched based for training status and VO_2max_, females were studied during the follicular phase of the menstrual cycle and study participants were subjected to a prolonged run of moderate intensity. Similar to the investigation described herein, these researchers observed increases in GH concentrations 15 min post-exercise in males, but not females, and increases in glucose and insulin levels 15 min post-exercise in females, with no changes observed in males. Although this study documented hormone concentrations 30 min post-exercise, similar responses were observed.

Contrary to the results of Tarnolpolsky et al, we observed post-exercise increases in FFAs in both genders with no differences noted between them. Tarnopolsky et al reported increased levels of circulating FFAs 15 min post-exercise only in males. This finding is surprising considering similar reports of increased post-exercise plasma concentrations of GH, a lipolytic hormone. However, it is possible that other hormones not measured in the present study but which influence substrate utilization (i.e. catecholamines), influenced this response. In addition, it is also possible that plasma FFAs in men did increase above that in women, but at time points other than those used in this study. Finally, gender differences existed in post-exercise lactate concentrations in our study, but not in that by Tarnopolsky and colleagues. This difference might be accounted for by the greater training volume in men (51 ± 4 miles/wk) compared to women (37 ± 1 miles/wk) in the present investigation. However, the magnitude of the difference in lactate levels was small and it is unclear whether this small difference at POST + 30 is either physiologically significant or indicative of a greater exercise stress for the females.

While the data herein are intriguing, it is important to consider study limitations in the context of study findings. Since studies in the present report were part of larger investigations [28,29] examining skeletal muscle protein turnover (SMPTO) responses to an endurance exercise bout, study measurements were done at varied time points over an extended period of time that coincided with muscle sampling. Therefore, the final measurement provides a "snapshot" of a delayed recovery response rather than another period of time further removed from completion of the exercise bout. As a result, caution must be used in extending these findings to the general population of competitive, healthy, endurance trained men and women. In addition, the time points used for assessing the post-exercise response during the earlier phase of recovery were later (15–20 min) than those used by other studies investigating similar measures. Although both genders were considered fit with no differences in VO_2peak _values when corrected for FFM, it is possible that the higher weekly mileage of the men contributed to some of the differences noted between genders. For example, the men most likely covered more distance (i.e., performed more work) during the run at 70% of their respective VO_2peak _and subsequently mobilized greater amounts of substrates during the exercise bout than the women. Similarly, while all parameters for exercise protocols were held constant between studies, the exercise bout may have been less stressful for the men. Finally, the small sample size for the present study likely impacted study findings.

## Conclusion

In summary, this study identified gender differences in the substrate and endocrine responses during the three-and-a-half hour recovery period from moderate intensity endurance exercise in trained runners while controlling for variables known to confound these responses (i.e. diet prior to measurements, training volume/history, and menstrual cycle phase in females). Our findings further document the existence of gender differences in the substrate and endocrine response following an endurance exercise bout. In addition, they help in characterizing the time course of these differences during a three-and-a-half hour recovery period, beginning 30 min following aerobic exercise. Future studies should investigate the overall hormonal mileu (i.e. anabolic vs catabolic vs null) between men and women during the extended recovery period post endurance exercise while feeding controlled diets of similar macronutrient content to determine the impact of these differences on glycogen storage, muscle recovery, and other important components of recovery from endurance exercise. Finally, research should also aim to identify the impact of differing diets on these post-exercise metabolic differences to better understand how habitual intake of various macronutrients might impact and enhance recovery in men and women.

## Abbreviations

CHO, carbohydrate; FFA, free fatty acids; GH, growth hormone; IGF-I, insulin-like growth factor I; IMTG, intramyocellular triglycerides; PRO, protein; REE, resting energy expenditure; VO2max, maximal oxygen consumption; VO2peak, peak oxygen consumption.

## Competing interests

This study was supported in part by the National Cattlemen's Beef Association (NCBA), the Gatorade Sports Science Institute (GSSI), and the University of Connecticut Research Foundation. The results of the present study do not constitute endorsement of the NCBA or the GSSI by the authors or by ISSN.

## Authors' contributions

LMV participated in the implementation of the study, assisted in carrying out the immunoassays, and drafted the manuscript. PCG conceived of the study, participated in its design and coordination, carried out the immunoassays, performed the statistical analyses, and helped draft the manuscript. MAP participated in the implementation of the study and assisted in carrying out the immunoassays. WFM participated in the implementation of the study and assisted in carrying out the immunoassays. NRR participated in the study design and helped draft the manuscript. All authors read and approved the final manuscript.
